# An algorithm for thoracic re-irradiation using biologically effective dose: a common language on how to treat in a “no-treat zone”

**DOI:** 10.1186/s13014-021-01977-1

**Published:** 2022-01-06

**Authors:** Eric D. Brooks, Xiaochun Wang, Brian De, Vivek Verma, Tyler D. Williamson, Rachel Hunter, Abdallah S. R. Mohamed, Matthew S. Ning, Xiaodong Zhang, Joe Y. Chang

**Affiliations:** 1 Department of Radiation Oncology, University of Texas MD Anderson Cancer Center, 1515 Holcombe Blvd, Houston, TX USA; 2 Departments of Radiation Physics, University of Texas MD Anderson Cancer Center, Houston, TX USA; 3 Department of Radiation Oncology, University of Florida Health Proton Therapy Institute, Jacksonville, FL USA; 4 Department of Radiation Oncology, Allegheny General Hospital, Pittsburgh, PA USA

**Keywords:** Re-irradiation, Biologically effective dose, Equivalent dose, Dosimetry, Stereotactic ablative radiotherapy, Stereotactic body radiation therapy, Lung cancer

## Abstract

**Background:**

Re-irradiation (re-RT) is a technically challenging task for which few standardized approaches exist. This is in part due to the lack of a common platform to assess dose tolerance in relation to toxicity in the re-RT setting. To better address this knowledge gap and provide new tools for studying and developing thresholds for re-RT, we developed a novel algorithm that allows for anatomically accurate three-dimensional mapping of composite biological effective dose (BED) distributions from nominal doses (Gy).

**Methods:**

The algorithm was designed to automatically convert nominal dose from prior treatment plans to corresponding BED value maps (voxel size 2.5 mm^3^ and α/β of 3 for normal tissue, BED_3_). Following the conversion of each plan to a BED_3_ dose distribution, deformable registration was used to create a summed composite re-irradiation BED_3_ plan for each patient who received two treatments. A proof-of-principle analysis was performed on 38 re-irradiation cases of initial stereotactic ablative radiotherapy (SABR) followed by either re-SABR or chemoradiation for isolated locoregional recurrence of early-stage non-small cell lung cancer.

**Results:**

Evaluation of the algorithm-generated maps revealed appropriate conversion of physical dose to BED at each voxel. Of 14 patients receiving repeat SABR, there was one case each of grade 3 chest wall pain (7%), pneumonitis (7%), and dyspnea (7%). Of 24 patients undergoing repeat fractionated radiotherapy, grade 3 events were limited to two cases each of pneumonitis and dyspnea (8%). Composite BED_3_ dosimetry for each patient who experienced grade 2–3 events is provided and may help guide development of precise cumulative dose thresholds for organs at risk in the re-RT setting.

**Conclusions:**

This novel algorithm successfully created a voxel-by-voxel composite treatment plan using BED values. This approach may be used to more precisely examine dosimetric predictors of toxicities and to establish more accurate normal tissue constraints for re-irradiation.

## Introduction

Thoracic cancers such as non-small cell lung cancer (NSCLC) are associated with relatively higher rates of locoregional recurrence (LRR) as compared to other malignancies; up to 1 in 6 patients will experience LRR after treatment for early-stage disease [1]. Management of LRR is a challenging task for clinicians, who must balance a need to potentially curable disease with elevated risks of toxicities in the setting of prior treatment. In particular, re-irradiation (re-RT) of intrathoracic disease represents a technically difficult circumstance for which there remains a paucity of high-quality prospective data [2]. This absence of evidence may lead reluctance among clinicians to utilize curative-intent re-RT. Additional data on re-RT safety may encourage clinicians to treatment more of these recurrences.

Owing to this lack of data, the dose/fractionation schemes for re-RT cases have remained heterogeneous and based primarily on qualitative review of the prior treatment plan by clinicians. Examination of the degree of dose distribution overlap is commonly implemented for this purpose, and re-RT has remained more art than science. However, studying re-RT safety and effectiveness in a consistent, scientifically rigorous, and generalizable way is treacherous for several reasons. First, dose/fractionation regimens between prior and repeat RT nearly always differ, and thus manual calculations of biologically effective doses (BEDs) and/or equivalent dose in 2 Gy fractions (EQD2) to various organs at risk (OARs) are often required to provide a “common language” such that the cumulative biologic tolerance as a whole can be estimated [2]. Research on this topic has been limited, and cumulative OAR tolerances remain elusive, not only because there have been few patients studied, but also because there is no streamlined way to evaluate this biologically effective exposure when heterogeneous regimens are used. This is especially true today when hypofractionated or stereotactic ablative radiotherapy (SABR; also known as stereotactic body radiation therapy [SBRT]) may be given for re-RT. These hypofractionated regimens have the advantage of safely escalating BED while also minimizing high-grade toxicities in an attempt to deliver durable tumor control for recurrent disease. While they are attractive options in the re-RT setting, the lack of safety data has made clinicians hesitant to use re-RT with definitive regimens.

There have been a number of smaller retrospective studies of hypofractionated or stereotactic re-RT, which have utilized a wide variety of dose/fractionation schemes [3–13]. In these publications, manual calculations of cumulative BEDs to thoracic OARs have been essential to the fidelity and quality of re-RT. These reports have helped to increase recognition of the feasibility of re-RT and have enabled important conclusions, such as the importance of avoiding < 5 fraction hypofractionation near central structures to mitigate hemorrhage risk, the importance of limiting dose to the proximal bronchial tree, esophagus, and heart, and the observation that delivery of high dose to previously irradiated non-functional lung does not confer unacceptable toxicity risk. Although the linear-quadratic model (LQM) may be inaccurate at high fractional doses [14, 15], which are often employed in these studies and in clinical settings, cumulative BED/EQD2 measurement remains the only common terminology amidst a multitude of dose/fractionation regimens. Despite the attempts by multiple authors to review smaller datasets of patients for thoracic re-RT, this research effort remains limited owing to a lack of more automated methods of assessing cumulative dose to OARs with a common metric, such as BED or EQD2.

Given the increasing popularity of use of re-RT, evaluation of composite BED or EQD2 distributions that allow for interpretation of OAR tolerances irrespective of dose/fractionation schemes is increasingly important. To address this knowledge gap and to provide a new tool for the study of re-RT, we developed a novel treatment planning algorithm tool that rapidly auto-converts nominal dose (Gy) from two RT plans into BED values for an individual patient. This tool does so for each anatomic voxel of the computed tomography (CT) dataset for each RT plan and generates a cumulative three-dimensional treatment plan with BED isodose lines (IDLs). This is done for each individual plan and then overlaid, upon which a map of anatomically-accurate composite BED can be created in order to visualize the areas of OARs exposed. Cumulative BED exposure can then be easily studied in relation to re-RT toxicity to promote the development of accurate cumulative OAR tolerances. Such tolerances can then be used during re-RT planning in the future. We employed this novel tool to study cumulative BED exposure for various OARs of patients undergoing thoracic re-RT at our center.

## Materials and methods

### Patient population and treatment details

The Institutional Review Board Committee at MD Anderson Cancer Center approved our request to review the medical records of these patients. The need for informed patient consent was waived, as this was a retrospective review and no identifiable patient information is included in this report. This study was conducted in accordance with the ethical standards of the Declaration of Helsinki and its later amendments.

After construction of the BED-conversion treatment planning algorithm tool we sought to validate its use in a cohort of patients who had received re-RT. We tested its utility in early-stage NSCLC patients who underwent re-RT (i.e., re-SABR for isolated lung parenchymal recurrence or repeat fractionated radiotherapy (RT)) for isolated LRR after initial SABR. Patients were extracted from an institutional SABR database of over 900 patients [16]. This specific population was chosen because the safety and efficacy of re-RT after primary SABR, for which few experiences have been published to date, has been deemed an active area of interest by the International Association for the Study of Lung Cancer [1].

Our institutional practice for the treatment of lung tumors in the re-RT setting has been previously reported. [1] All patients in the current study received either: (1) 50 Gy/4 fractions (fx) followed by 70 Gy/10fx, (2) 50 Gy/4fx followed by 50 Gy/4fx, (3) 70 Gy/10fx followed by 70 Gy/10fx, or (4) 50 Gy/4fx followed by 60 Gy/30fx. Doses were typically prescribed to the 70–90% IDL covering the planning treatment volume (PTV). For intensity-modulated radiotherapy (IMRT) or volumetric modulated arc therapy (VMAT), an integrated boost to the gross tumor volume (GTV) was typically used to generate a high-dose region. Given this approach, dose distribution was inherently inhomogeneous; target coverage and OAR sparing were prioritized over homogeneity.

### Plan deformation

All CT datasets from previous treatment courses were exported to Velocity software for dose (Gy) distribution deformation (VelocityAI 3.0.1, Velocity Medical, Atlanta, GA). Within Velocity, one of the treatment planning CTs was selected as the reference on which the other plan’s nominal dose distribution (Gy) would be deformed. As such, the selected reference CT acted to hold all independent dose distributions for each plan, which were overlaid but not yet summed. The selection of the reference RT course was arbitrary; the deformation process is designed to address anatomical differences.

**Fig. 1:**
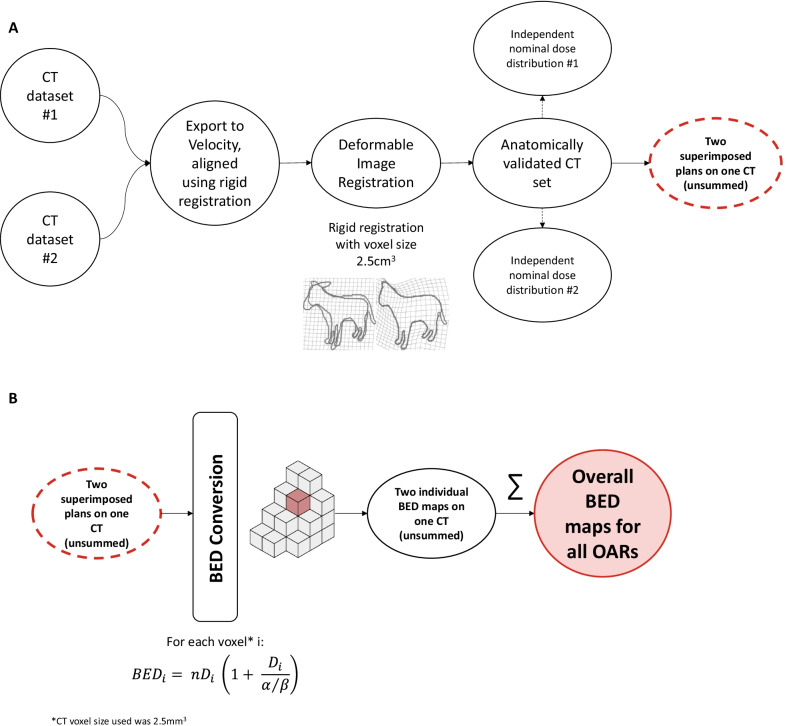
Workflow schematic for dose summation of two plans with disparate dose and fractionation schedules. **a** Workflow for image deformation for the original radiotherapy (CT #1) and reirradiation (CT #2) dataset plans. Both CT datasets were rigidly registered to bone followed by deformable image registration using 2.5 mm^3^ voxels. Since both CT sets belonged to the same patient, but at different time points, the choice of which acted as the reference CT was arbitrary. Once anatomically validated, individual dose distribution was overlaid on each CT #1 and #2 dataset. To validate that the final registration was accurate, a manual inspection was performed. In addition, we quantified organ at risk (OAR) doses for the registered/deformed image set and assured that the doses did not change. This indicated that registration preserved the OAR structure and distribution. Thus, after the initial registration process, two plans were superimposed but not yet converted to BED or summed but were anatomically aligned. **b** Workflow for the registered image sets then included conversion of physical dose of each individual plan into a corresponding BED plan. This was done using the automated algorithm for each data set (CT #1 and #2) at each 2.5 mm^3^ voxel. After BED conversion for each plan, the BED isodose lines for each plan were then summed to generate the composite BED dose exposed by each OAR. Thus, a composite BED isodose map was auto-generated for each patient who had received initial SABR followed by reirradiation. Composite BED exposure for each OAR was then assessed in terms of subsequent re-RT toxicity with the aim of developing potential dose thresholds

During the deformation process, rigid registration was first performed to align the CTs according to bony structures. Then, deformable image registration was carried out between the two CTs to obtain the transformation matrix, which was used to deform the nominal distribution (Gy) onto the reference CT. This produced two independent nominal dose distributions (Gy) on a single anatomically validated CT set, one nominal dose distribution from the original reference CT and one nominal dose distribution from the CT that was deformed (Gy). Subsequently, two plans were superimposed on one CT (unsummed), (Fig. 1a). The voxel size used for deformation was 2.5 mm^3^.

### BED conversion

The LQM was utilized to convert the nominal physical doses (Gy) to BED distributions in a voxel-by-voxel manner for each of the two individual data sets. For each voxel *i*, the BED was calculated as:

$${BED}_{i}= n{D}_{i} \left(1+ \frac{{D}_{i}}{\alpha /\beta }\right)$$

where n is the number of fractions, D_i_ is the physical fractional dose, and α/β is the OAR-specific ratio (designated as 3 for normal tissues). Although we selected an α/β of 3 for simplicity given our interest in normal tissue tolerance, it should be noted that any α/β value could theoretically be used. Since this study aimed to assess normal tissue toxicity risk, evaluation of BED for tumor voxels was not performed. The individual BED maps from each of the two plans were then subsequently summed to yield an overall BED map for all OARs. A workflow of the algorithm process is shown in Fig. 1b.

### BED summation and OAR evaluation

Converting dose (Gy) to BED allowed us to account for nonlinear biological response to differing dose per fraction, as BED distributions are additive according to the LQM. Thus, this novel process allowed the nominal distributions (Gy) from separate courses of treatment to be converted and summed to quantify the cumulative BEDs for each voxel in each OAR. The BED dose distribution was calculated by converting the nominal dose distribution (Gy) using an institutionally developed Python script. All BED dose distributions from the two different treatment courses for each patient were imported on the reference CT datasets in the Eclipse treatment planning system (Varian Medical Systems, Palo Alto, CA) and added together to obtain the cumulative BED dose distributions using the plan sum feature of the Eclipse system. The cumulative BED distributions to each OAR from both plans were then evaluated.

### Statistical analysis

The Kaplan–Meier method and life tables were used to evaluate overall survival (OS), which was calculated from completion of re-RT to death from any cause. Treatment-related toxic effects were scored with the Common Terminology Criteria for Adverse Events (CTCAE), version 4.0. Reporting of other continuous and categorical data is descriptive and comparison between groups was not indicated. Data were analyzed with SPSS, version 21.0 (IBM Corp, Armonk, NY).

## Results

**Fig. 2:**
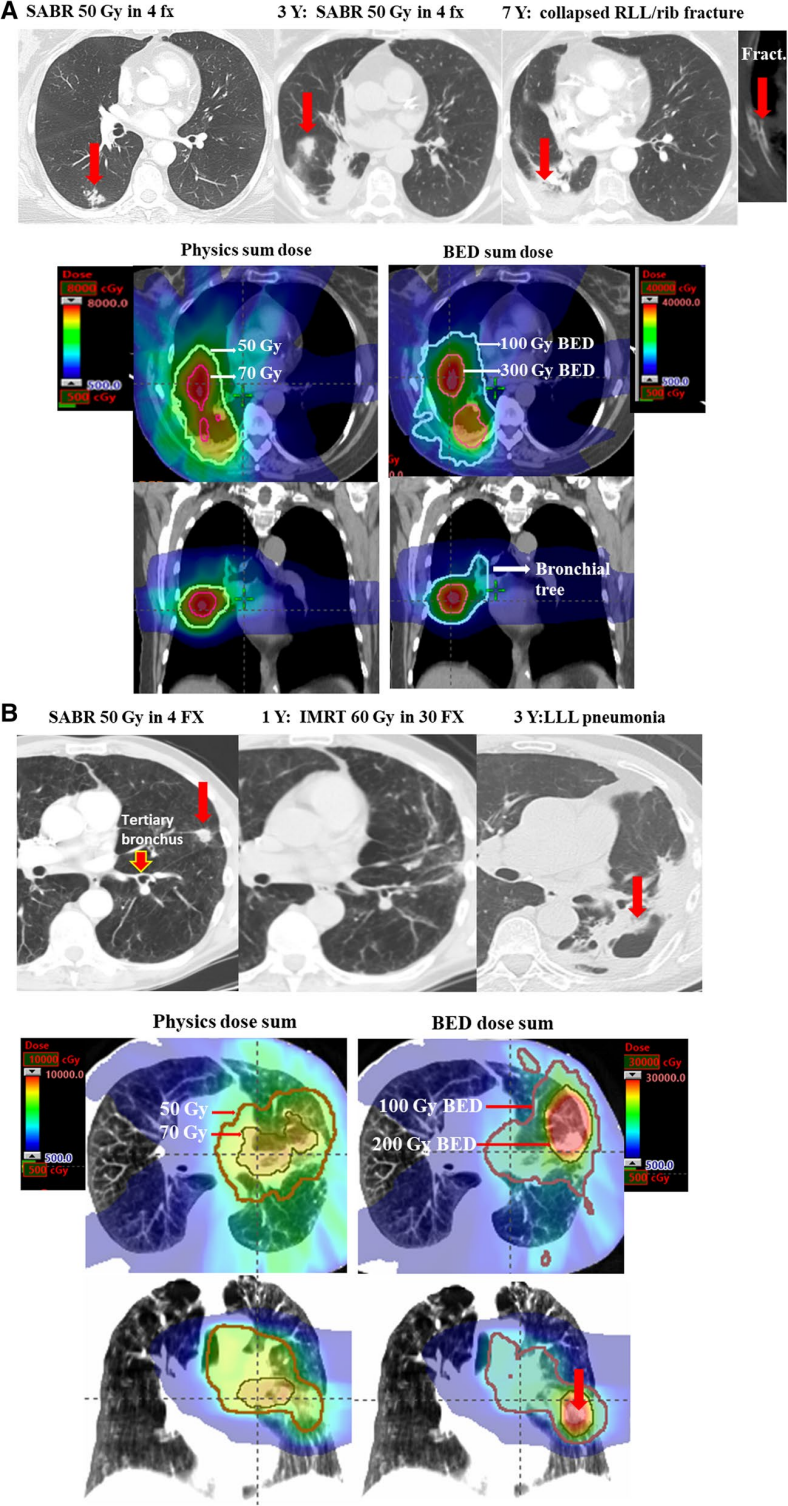
Examples of dose conversions for two patients treated with re-RT. **a** Example of physical dose to BED_3_ conversion for a 61-year-old patient treated with 50 Gy in 4 fractions to a right lower lobe T1N0 NSCLC lesion in 2007. The patient did well for three years but then experienced isolated local recurrence in the same lobe. He was treated to this nearby area using 50 Gy in 4 fractions. The figure depicts how the nominal doses in Gy for each of this patient’s individual plans were separately converted to their corresponding BED_3_ values. In this conversion, the patient’s two plans each had 2.5 mm^3^ voxels converted to corresponding BED_3_ values. This voxel-by-voxel conversion enabled an accurate anatomic and volumetric depiction of BED dose throughout the plans and organs at risk. The two plans were then summed to generate a BED composite, shown here. The patient developed symptomatic right rib fracture, correlated with BED_3_ of 100 Gy but less than 50 Gy by simple summation of the physical dose. The patient also developed shortness of breath requiring supplemental oxygen that may have been precipitated by RLL collapse, correlated with BED_3_ > 100 Gy in the right bronchial tree. **b** Example of physical dose to BED_3_ conversion for a patient who underwent SABR followed by IMRT. This 77-year-old patient received 50 Gy in 4 fractions for left upper lobe Stage I NSCLC, and 6 months later developed left hilar and mediastinal nodal recurrence which was treated with concurrent chemoradiotherapy to 60 Gy in 30 fractions with simultaneous integrated boost of gross disease to 66 Gy. The patient developed partial collapse of the left lower lobe and episodes of pneumonia, requiring supplemental oxygen. The damage to the left lower lobe tertiary bronchial tree is correlated with a BED_3_ dose sum of 200 Gy, but less than 70 Gy by simple summation of the nominal doses

Composite treatment plans using BED distributions were successfully generated with appropriate anatomic accuracy (Fig. 1). This, in turn, enabled composite BED_3_ evaluation of several OARs, such as the spinal cord, proximal tracheobronchial tree, great vessels, brachial plexus, chest wall, heart, esophagus, and total lung. Two representative cases of the physics Gy-to-BED_3_ conversion using the re-RT planning CT are presented in Fig. 2.

In total, 38 patients received initial SABR followed by re-SABR (*n* = 14) or fractionated RT with chemotherapy (*n* = 24). Characteristics of the population are shown in Table 1. Notably, all patients had T1-2N0 NSCLC at initial diagnosis, 71% of re-SABR cases and 67% of chemoradiation (CRT) subjects had adenocarcinoma, and recurrence was confirmed by biopsy in 50% of re-SABR patients and 100% of CRT patients. The remaining recurrences were confirmed by dedicated diagnostic imaging. The median time to recurrence from initial SABR was 20 months for re-SABR patients and 16 months for CRT after SABR. The median follow-up time from the date of completion of re-RT was 36 months for re-SABR patients and 18 months for CRT after SABR patients. The estimated 3-year OS from the time of recurrence was 63% for re-SABR patients and 35% for CRT after SABR patients.

**Table 1:** Patient demographics

Characteristics	Patients with re-SABR (n = 14)	Patients with CRT after SABR (n = 24)
Age (at time of recurrence), median (range)	74 (57–84)	70 (49–85)
Sex		
Male Female	8 (57%) 6 (43%)	14 (58%) 10 (42%)
ECOG (at time of recurrence)		
0 1 2 3	0 (0%) 13 (93%) 1 (7%) 0 (0%)	4 (17%) 15 (63%) 5 (21%) 0 (0%)
Tumor stage (at initial presentation)		
T1 T2	12 (86%) 2 (14%)	22 (92%) 2 (8%)
Histology		
Adenocarcinoma Squamous Other	10 (71%) 4 (29%) 0 (0%)	16 (67%) 7 (29%) 1 (4%)
EBUS performed initially	10 (71%)	20 (83%)
Recurrence confirmed		
Biopsy PET-CT CT	7 (50%) 4 (29%) 3 (21%)	24 (100%) 0 (0%) 0 (0%)
Median time to recurrence from 1st SABR, mo (range)	20 (3–60)	16 (5–54)
Median follow-up time from the time of re-RT, mo (IQR)	36 (19–45)	18 (8–38)
OS after re-RT (95% CI)		
1-year rate, % 3-year rate, % 5-year rate, %	86% (54–96%) 63% (32–83%) 54% (24–76%)	70% (47–84%) 35% (17–54%) 16% (3–36%)

Overall, there were no instances of grade 4–5 events in any patient who underwent re-RT. Of the re-SABR population, the overall rate of higher-grade toxicities was low; there was one case each of grade 3 chest wall pain (7%, Fig. 2a), pneumonitis (7%, Fig. 2a), and dyspnea (7%). Other grade 2 events included brachial plexopathy (n = 1, 7%), rib fracture (n = 3, 21%), chest wall pain (n = 1, 7%), pneumonitis (n = 1, 7%), and dyspnea (n = 3, 21%). It was not possible to determine whether dyspnea was attributable to natural progression of underlying lung disease (e.g., chronic obstructive pulmonary disease) or re-treatment. Table 2 lists BED_3_ dosimetric parameters for OARs in all patients who experienced these toxicities.

**Table 2:** Dosimetric characteristics of patients experiencing selected grade 2–3 toxicities from repeat SABR and CRT

Organ at risk (OAR)	Grade and number (%) of re-SABR toxicity	Composite BED_3_ or % corresponding to Re-SABR toxicity, mean (range)	Grade and number (%) of re-CRT toxicity	Composite BED_3_ or % corresponding to toxicity, mean (range)
Brachial plexus				
D_max_ D_0.2 cc_	Grade 2 brachial plexopathy, n = 1 (7%)	144 Gy 123 Gy	-	-
Chest wall				
D_max_ D_30cc_ D_50cc_	Grade 2 rib fracture, n = 3 (21%)	370 Gy (288–432) 225 Gy (166–339) 187 (122–308)	Grade 2 rib fracture, n = 2 (8%)	403 Gy (353–452) 209 Gy (175–243) 139 (100–177 Gy)
Chest wall				
D_max_ D_30cc_ D_50cc_	Grade 2 chest wall pain, n = 1 (7%)	411 Gy 107 Gy 83 Gy	Grade 2 chest wall pain, n = 3 (13%)	386 Gy (353–452) 243 Gy (175–310) 177 Gy (100–255)
D_max_ D_30cc_ D_50cc_	Grade 3 chest wall pain, n = 1 (7%)	390 Gy 339 Gy 308 Gy	-	-
Total Lung				
Mean D_max_ V5 V20 V35 Mean D_max_ V5 V20 V35	Grade 2 pneumonitis, n = 1 (7%) Grade 3 pneumonitis, n = 1 (7%)	15 Gy 451 Gy 32% 13% 10% 35 Gy 481 Gy 32% 23% 20%	Grade 2 pneumonitis, n = 1 (4%) Grade 3 pneumonitis, n = 2 (8%)	29 Gy 382 Gy 67% 33% 25% 21 Gy (20–22) 391 (332–449) 36% (34–37) 24% (23–25) 19% (18–19)
Total lung				
Mean D_max_ V5 V20 V35	Grade 2 dyspnea, n = 3 (21%)	24 Gy (11–33) 487 Gy (377–622) 49% (23–77) 27% (11–45) 17% (7–25)	Grade 2 dyspnea, n = 2 (8%)	33 Gy (32–34) 444 Gy (362–526) 62% (55–68) 36% (30–41) 28% (24–31)
Mean D_max_ V5 V20 V35	Grade 3 dyspnea, n = 1 (7%)	35 Gy 481 Gy 32% 23% 20%	Grade 3 dyspnea, n = 2 (8%)	26 Gy (22–29) 416 Gy (382–449) 52% (37–67) 28% (23–33) 22% (19–25)

CRT patients also demonstrated an acceptably low rate of adverse effects with no grade 4–5 toxicities reported. The only grade 3 events were two cases of pneumonitis (8%, Fig. 2b) and dyspnea (8%). The grade 2 toxicities were rib fracture (n = 2, 8%), chest wall pain (n = 3, 13%), pneumonitis (n = 1, 4%), and dyspnea (n = 2, 8%). Again, ascertainment of the cause of dyspnea was not possible and may have been related to treatment, natural progression of underlying lung disease, or other factors. Dosimetric parameters in BED_3_ for patients who experienced these toxicities are listed in Table 2.

Cumulative BED_3_ doses for patients who experienced grade 2–3 events are reported in Table 2. Cumulative BED_3_ doses for the entire patient cohort are presented in Table 3, including details of cumulative thoracic OAR BED_3_ parameters associated with toxicities not exceeding grade 3.

**Table 3:** BED_3_ and toxicity characteristics for all patients receiving re-irradiation

Organ at risk	BED_3_ composite, mean (range)	Re-SABR toxicity, n (%) (n = 14)	CRT after SABR toxicity, n (%) (n = 24)	Total toxicity, n (%) (n = 38)
Spinal cord	Max: 40 Gy (5 Gy- 91 Gy) D1cc: 34 Gy (1 Gy- 63 Gy)	0 (0%)	0 (0%)	0 (0%)
Trachea	-	0 (0%)	0 (0%)	0 (0%)
Proximal bronchial tree	Max: 133 Gy (7 Gy- 253 Gy) D1cc: 108 Gy (2 Gy- 220 Gy)	0 (0%)	0 (0%)	0 (0%)
Aorta	Max: 120 Gy (22 Gy – 332 Gy) D1cc: 105 Gy (15 Gy – 242 Gy)	0 (0%)	0 (0%)	0 (0%)
Pulmonary artery	Max: 114 Gy (2 Gy – 395 Gy) D1cc: 101 Gy (1 Gy – 284 Gy)	0 (0%)	0 (0%)	0 (0%)
Superior vena cava	Max: 89 Gy (6 Gy – 184 Gy) D1cc: 77 Gy (40 Gy – 162 Gy)	0 (0%)	0 (0%)	0 (0%)
Brachial plexus	Max: 16 Gy (0 Gy – 145 Gy) D0.2 cc: 13 Gy (0 Gy – 123 Gy)	Brachial plexopathy G2 n = 1 (7%)	Brachial plexopathy n = 0 (0%)	Brachial plexopathy G2 n = 1 (3%)
Chest wall	Max: 320 Gy (62 Gy – 568 Gy) D30cc: 145 Gy (39 Gy – 339 Gy) D50cc: 116 Gy (33 Gy – 308 Gy)	Dermatitis G1 n = 1 (7%) CW pain G1 n = 1 (7%) G2 n = 1 (7%) G3 n = 1 (7%) Rib fracture G2 n = 3 (21%)	Dermatitis G1 n = 5 (21%) G2 n = 1 (4%) CW pain G1 n = 2 (8%) G2 n = 3 (13%) Rib fracture G2 n = 2 (8%)	Dermatitis G1 n = 6 (16%) G2 n = 1 (3%) CW pain G1 n = 3 (8%) G2 n = 4 (11%) G3 n = 1 (3%) Rib fracture G2 n = 5 (13%)
Esophagus	Mean: 24 Gy (1 Gy – 67 Gy) Max: 94 Gy (5 Gy – 218 Gy) D30cc: 73 Gy (1 Gy – 190 Gy) D50cc: 65 Gy (1 Gy – 174 Gy)	Fatigue G1 n = 5 (36%) G2 n = 1 (7%) Esophagitis n = 0 (0%)	Fatigue G1 n = 10 (42%) G2 n = 3 (13%) Esophagitis G1 n = 11 (46%) G2 n = 4 (17%)	Fatigue G1 n = 15 (39%) G2 n = 4 (11%) Esophagitis G1 n = 11 (29%) G2 n = 4 (1%)
Heart	Mean: 11 Gy (0 Gy – 58 Gy) Max: 98 Gy (1 Gy – 280 Gy) D5cc: 64 Gy (0 Gy – 132 Gy) D40cc: 36 Gy (0 Gy –111 Gy)	0 (0%)	0 (0%)	0 (0%)
Total lung	Mean: 23 Gy (6 Gy – 50 Gy) Max: 434 Gy (100 Gy – 729 Gy) V5Gy: 43.6% (16.2% – 86.9%) V20Gy: 24.6% (8.7% – 46.3%) V35Gy: 18.6% (3.5% – 39.7%)	Dyspnea G1 n = 7 (50%) G2 n = 3 (21%) G3 n = 1 (7%) Cough G1 n = 6 (43%) Pneumonitis G2 n = 1 (7%) G3 n = 1 (7%)	Dyspnea G1 n = 2 (8%) G2 n = 2 (8%) G3 n = 2 (8%) Cough G1 n = 8 (33%) G2 n = 1 (4%) Pneumonitis G1 n = 6 (25%) G3 n = 2 (8%)	Dyspnea G1 n = 9 (24%) G2 n = 5 (13%) G3 n = 3 (8%) Cough G1 n = 14 (37%) G2 n = 1 (3%) Pneumonitis G1 n = 6 (16%) G2 n = 1 (3%) G3 n = 3 (8%)

Lastly, for each case of grade 3 toxicity (n = 5), we evaluated the predicted doses to the corresponding OARs based on simple summation of the nominal doses versus the BED_3_ dose sum. It was noted that, in two (40%) instances, the former reported lower doses to OARs as compared to the latter (Fig. 2), suggesting that BED_3_-based dose sum planning may better predict higher-grade adverse events. These data suggest that BED_3_ dose sums may have higher utility in establishing dose-volume constraints as compared to nominal dose sums and may allow clinicians to anticipate long-term re-RT toxicities in a more accurate fashion.

## Discussion

Re-irradiation is a technically challenging undertaking for which few standardized approaches exist. This is in part due to the lack of a common language to assess dose tolerance in relation to toxicity in the re-RT setting. To better address this knowledge gap and provide new tools for studying and developing thresholds for re-RT, we developed a novel algorithm that allows for anatomically accurate three-dimensional mapping of composite BED distributions from nominal doses. This approach provides a practical framework to assist clinicians in deciding upon dose/fractionation schemes for this population. Further, it may allow dose constraints to be generated based on the BED an OAR has already received. Preliminary analysis of each grade 3 event indicates that BED_3_-based planning may better anticipate higher-grade toxicities and enable clinicians to more confidently develop treatment plans that minimize morbidity. The success of this platform in our cohort of thoracic patients receiving re-RT is noteworthy and analogous studies in other disease sites are encouraged.

The primary impetus for this investigation was the lack of semi-automated and standardized approaches to re-RT, especially given the heterogeneity of dose/fractionation schemas and the inability to three-dimensionally visualize composite BED distributions. This study demonstrates that implementation of an algorithm to map these values using BED IDLs is valuable in accurately evaluating dosimetric predictors of toxicities, as well as in practically ascertaining OAR dose tolerances in the re-RT setting. In this manner, if the BED to an OAR is calculated from the initial treatment course, clinicians can calculate the additional BED an OAR can tolerate during a re-RT course with ease. Then, by using the number of planned fractions for re-RT, the maximum safe nominal dose to that OAR can be computed. This approach can be used to inform safe dose/fractionation schemes for these challenging cases.

This study also adds important information to the literature regarding the safety and efficacy of re-SABR for isolated LRRs from early-stage NSCLC patients receiving initial SABR. It is essential to determine dose constraints in this setting, since few investigations [5, 9, 12] have examined dosimetric predictors of higher-grade re-RT toxicities. Existing reports have suggested that SABR or hypofractionated re-RT near the mediastinum can lead to bleeding events, and that cumulative dose to the great vessels not exceeding 120 Gy may reduce this risk [17, 18]. One report of conformal thoracic re-RT suggested a higher grade 3 esophagitis risk with cumulative EQD2 of 75 Gy or higher [19] and another found correlations between maximum point dose and V60 of the re-RT course with esophagitis [20]. Data regarding appropriate dose constraints for the proximal bronchial tree in the re-RT setting are more limited; two reports have suggested that an EQD2 maximum point dose of < 80 Gy should be considered [21, 22]. These and other publications examining salvage treatment for this population have illustrated several overarching conclusions [1]. First, locoregional failures are indeed potentially curable with a variety of management approaches, such as salvage surgery, re-irradiation, and thermal ablation. Second, patients having received salvage therapy can experience relatively long post-salvage disease-free and overall survival, especially when compared to unsalvaged subjects. Third, salvage (especially nonoperative) therapies are associated with a relatively low incidence of higher-grade toxicities. This notion is important because salvage therapy in the previously irradiated lung can theoretically cause serious complications. Salvage re-SABR for this population is expected to gain popularity [1] and use of a reliable platform to better standardize the re-RT process is attractive.

Although clinically reassuring, the low rate of grade 2 + toxicities in this population precluded robust multivariable analysis to examine whether there were dosimetric predictors independently associated with adverse events in this setting. However, the algorithm presented in the current study may enable such conclusions to be drawn in future analyses. We have presented individualized dosimetric data in Tables 2 and 3 in an effort to guide clinicians based upon our experience. However, it should be acknowledged that without formal statistical comparison, no definite association can be made between a particular composite OAR dose and the development of adverse events. As such, these values may provide guidance but should not be implemented as validated thresholds used in clinical practice in light of the small sample size and low event rate. Nevertheless, this work represents an important advance in a time when increasingly complex dose/fractionation regimens are being used for which nominal interpretation remains difficult. We encourage other centers to use similar approaches to quickly and reliably evaluate potential re-RT toxicity and to aid in the development of re-RT constraints. Recent guidelines have been proposed to address dose constraints in the reirradiation setting; their evolution and acceptance would be aided by robust data utilizing the approach put forward in the current study [23, 24].

There are several limitations of this study in addition to its retrospective nature and small sample size. First, no adjustment was made for the time interval between initial and repeat irradiation owing to an absence of precise data that characterizes normal tissue repair over time. This may be incorporated in future iterations of this algorithm as higher quality data emerge. Second, we assumed that OARs had an α/β of 3, which may be an oversimplification. However, the algorithm allows for adjustment based on any desired α/β ratio and differential values may be incorporated. Third, this population may not be reflective of a “generic” post-SABR re-irradiation population, given that the threshold to perform salvage treatment varies by clinician and institution. Fourth, plan deformation overlay as well as algorithm construction and execution are inherently imperfect processes and thus cannot reflect the BED received by OARs with absolute certainty. Fifth, although proton re-RT is becoming an increasingly common approach [25], this platform was only studied in the setting of photon re-RT; nevertheless, the algorithm does allow for input of mixed photon-proton irradiation courses. Sixth, receipt of systemic therapy was not standardized for these patients; we acknowledge that BED tolerances may differ when stratified for delivery of concurrent systemic therapy and this indeed necessitates further investigation. Lastly, there may be differences in calculated OAR BED tolerances for patients receiving conventionally fractionated RT vs. a repeat hypofractionated course for the second course of RT, which requires further investigation. Future studies should validate the findings herein and further refine the construction of a framework to assist clinicians in developing safe, effective treatment plans.

## Data Availability

The data that support the findings of this study are available from the corresponding author, JYC, upon reasonable request.

## Abbreviations

Re-RT: Reirradiation
Gy: Gray
BED: Biologically effective dose
EQD2: Equivalent dose at 2 Gy/fraction
SABR: Stereotactic ablative radiotherapy
SBRT: Stereotactic body radiation therapy
CRT: Chemoradiation
NSCLC: Non-small cell lung cancer
LRR: Locoregional recurrence
OAR: Organ at risk
CT: Computed tomography
IDL: Isodose line
LQM: Linear-quadratic model
CTCAE: Common terminology criteria for adverse events
OS: Overall survival
